# Effects of IL-10 and glucose on expression of OPG and RANKL in human periodontal ligament fibroblasts

**DOI:** 10.1590/1414-431X20154324

**Published:** 2016-04-08

**Authors:** L. Zhang, Y. Ding, G.Z. Rao, D. Miao

**Affiliations:** 1 Stomatology Hospital, Xi'an Jiaotong University College of Medicine, Xi'an, China; 2 Wuxi Mental Health Center of, Nanjing Medical University, Wuxi, China

**Keywords:** Human periodontal ligament fibroblasts, Interleukin-10, Glucose, Osteoprotegerin, Receptor activator of nuclear factor-κB ligand

## Abstract

The effects of interleukin-10 (IL-10) and glucose on mRNA and protein expression of osteoprotegerin (OPG), and its ligand, receptor activator of nuclear factor-κB ligand (RANKL), were investigated in human periodontal ligament fibroblasts (HPDLFs). Primary HPDLFs were treated with different concentrations of IL-10 (0, 1, 10, 25, 50, and 100 ng/mL) or glucose (0, 5.5, 10, 20, 30, and 40 mmol/L). Changes in mRNA and protein expression were examined using the reverse-transcription polymerase chain reaction (RT-PCR) and Western blot analysis, respectively. After IL-10 treatment, mRNA and protein levels of OPG were increased, while mRNA and protein levels of RANKL were decreased (P<0.05), both in a concentration-dependent manner. Glucose stimulation had the opposite concentration-dependent effect to that of IL-10 on OPG and RANKL expression. IL-10 upregulated OPG expression and downregulated RANKL expression, whereas high glucose upregulated RANKL and downregulated OPG in HDPLFs. Abnormal levels of IL-10 and glucose may contribute to the pathogenesis of periodontal disease.

## Introduction

Osteoprotegerin (OPG) and its ligand, receptor activator of nuclear factor-κB ligand (RANKL), are critical factors in regulating the differentiation and maturation of osteoclasts, as well as bone resorption (1). The equilibrium between OPG and RANKL activity has an essential role in the homeostasis of bone metabolism. In the pathological process of periodontal disease, the OPG/RANKL equilibrium is disrupted, leading to increased bone resorption (2,3). Human periodontal ligament fibroblasts (HPDLFs) are the primary cell type in the periodontal ligament and they contribute to the integrity of the periodontium. HPDLFs express both OPG and RANKL, affecting the formation of osteoclasts by modulating the OPG/RANKL equilibrium (4).

Multiple cytokines, which have different effects on the expression of OPG and RANKL, are involved in the pathogenesis of periodontal disease (5). Interleukin (IL)-10 is an important anti-inflammatory cytokine. It has been demonstrated that lack of IL-10 leads to more severe periodontal inflammation and further accelerates bone loss (6). However, the regulatory effect of IL-10 on the expression of OPG and RANKL has not yet been defined.

Diabetes is an endocrine and metabolic disorder that is caused by aberrant insulin function, leading to systemic bone metabolism disorders and osteoporosis (7). Diabetic patients with periodontal disease have severe periodontal destruction, progressive alveolar bone loss, and a poor prognosis (8,9). These studies indicate that diabetes-associated hyperglycemia may contribute to the progression of periodontal disease. Furthermore, it has been suggested that low levels of IL-10 correlate with the pathogenesis of diabetes (10). The aim of this study was to explore the influence of IL-10 and elevated glucose concentrations on the expression of OPG and RANKL in HPDLFs.

## Material and Methods

### Primary culture of HPDLFs

This study was approved by the Human Ethics Committees of Xi'an Jiaotong University (Xi'an, China; approval number XAJTU-22). Written informed consent was obtained from all study participants. HPDLFs were isolated from six clinically normal premolar teeth during orthodontic treatment (11). The teeth were placed in sterile D-Hanks solution containing ampicillin (200 µg/mL) and sulfuric streptomycin (200 µg/mL), and washed. Periodontal tissues were scraped from the middle one-third of the teeth roots, cut into pieces in Dulbecco's modified Eagle's medium (DMEM) without fetal bovine serum (FBS), and then centrifuged at 800 *g* for 5 min followed by supernatant removal. The periodontal tissue pellets were suspended in DMEM with 20% FBS, transferred to flasks coated by semi-dry FBS, and cultured under 5% CO_2_, 37°C, and saturated humidity (by inversion of the flasks). After 4 h of culture, 2 mL of DMEM with 20% FBS was added to the medium, and the flask was turned over gently for continued culturing. The medium containing 20% FBS was changed every 2–3 days. Cells from the fifth passage were seeded on coverslips in 12-well plates at a density of 10^4^ cells/mL until 60%–70% confluence. After experimental treatments, the cells were stained with hematoxylin and eosin (H&E), and cytochemistry analysis for vimentin and keratin was performed.

### IL-10 and glucose treatment

HPDLFs were harvested, and then cultured in 25-mL flasks at a density of 5.0×10^5^ cells/mL in DMEM with 20% FBS until cells adhered to the flask at 80% confluence. The culture medium was replaced with DMEM without FBS for 24 h before experiments. HPDLFs were cultured in DMEM with 6 different concentrations of IL-10 and glucose for 24 h. The concentrations of IL-10 were 0, 1, 10, 25, 50, and 100 ng/mL (12), and the concentrations of glucose were 0, 5.5, 10, 20, 30, and 40 mmol/L (13).

### RT-PCR analysis

Total RNA was isolated from HPDLFs using Trizol kits according to the manufacturer's instructions. The absorbance at 260 nm (OD260) and 280 nm (OD280) was measured, and the purity of RNA was determined by the OD260/OD280 ratio. cDNA was generated from total RNA by RT-PCR. The PCR primers for OPG, RANKL and β-actin are listed in Table 1. PCR cycles were performed as follows: initial denaturation at 94°C for 3 min, followed by 35 cycles of denaturation at 94°C for 15 s, annealing for 30 s at the indicated temperatures, and extension for 60 s at 72°C. The annealing temperature for OPG, RANKL, and β-actin was 55°C, 58°C, and 55°C, respectively. PCR products were visualized by agarose gel electrophoresis. The grey-scale value of each band was measured by the gel image analyzing system.

**Table:**
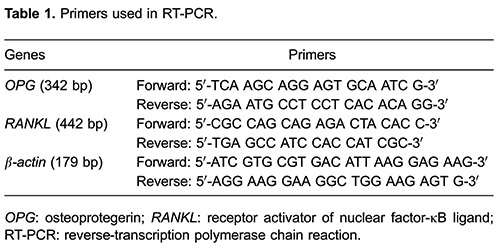

### Western blot analysis

Cells were lysed with radio-immunoprecipitation assay (RIPA) buffer and protein concentrations were measured by the bicinchoninic acid (BCA) assay. Samples containing an equal amount of protein mixed with sample buffer were loaded into each well, resolved by 10% SDS-PAGE, and electroblotted onto polyvinylidene difluoride membranes. The membranes were blocked for 1 h at room temperature and incubated with primary antibodies at 4°C overnight, followed by appropriate horseradish peroxidase-conjugated secondary antibodies for 1 h at room temperature. After washing, the membranes were developed using a West-Pico ECL kit (Pierce Chemical Co., USA). The following specific primary antibodies were used: mouse anti-OPG, anti-RANKL, and anti-glyceraldehyde-3-phosphate dehydrogenase (GAPDH) antibodies (Santa Cruz Biotechnology, USA).

### Statistical analysis

Data were analyzed by one-way analysis of variance, followed by Tukey's multiple comparison. Results are reported as means±SD. Statistical analyses were performed using the SPSS 13.0 software package (SPSS Inc., USA). P-values of less than 0.5 were considered to be statistically significant.

## Results

### Cell morphology

**Figure 1:**
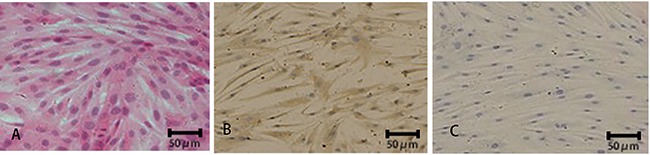
. Characterization of human periodontal ligament fibroblasts (HPDLFs). H&E staining (*A*) and immunocytochemical staining for vimentin (*B*) and keratin (*C*) were performed in HPDLFs. Representative images are shown.

Under the light microscope, H&E staining revealed that HPDLFs were spindle-shaped with several protrusions. Plasma was stained pink with round or oval nuclear centers stained purple (Figure 1A). Immunocytochemistry showed positive cytoplasmic staining for vimentin (Figure 1B), but not keratin (Figure 1C).

### Effect of IL-10 and glucose on OPG and RANKL mRNA expression

**Figure 2:**
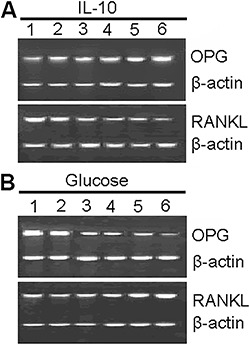
Effects of IL-10 (*A*) and glucose (*B*) at different concentrations on the mRNA expression of osteoprotegerin (OPG) and receptor activator of nuclear factor-κB ligand (RANKL) in human periodontal ligament fibroblasts. *Panel A*, *lanes 1-6*: cells treated with interleukin-10 (IL-10) at 0, 1, 10, 25, 50, and 100 ng/mL, respectively. *Panel B*, *lanes 1-6*: cells treated with glucose at 0, 5.5, 10, 20, 30, and 40 mmol/L, respectively.

The effects of IL-10 and glucose on OPG and RANKL mRNA expression were determined by RT-PCR analysis (Figure 2). Table 2 shows the densitometric analysis of OPG and RANKL mRNA levels normalized against β-actin. Compared with untreated cells, IL-10 treatment upregulated OPG mRNA expression and downregulated RANKL mRNA expression (P<0.05), with both changes occurring in a concentration-dependent manner. At normal physiological concentration (5.5 mmol/L), glucose had only a mild effect on mRNA expression of OPG and RANKL. However, at higher concentrations (10-40 mmol/L), glucose reduced mRNA levels of OPG and increased mRNA levels of RANKL (P<0.05 for both).

**Table:**
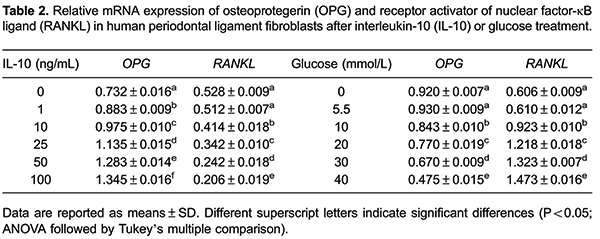

### Effect of IL-10 and glucose on OPG and RANKL protein expression

**Figure 3:**
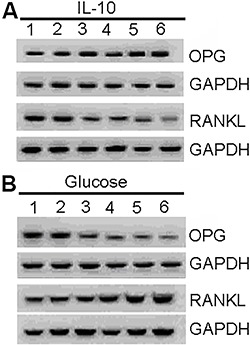
Effects of IL-10 (*A*) and glucose (*B*) at different concentrations on the protein expression of osteoprotegerin (OPG) and receptor activator of nuclear factor-κB ligand (RANKL) in human periodontal ligament fibroblasts. *Panel A*, *lanes 1-6*: cells treated with interleukin-10 (IL-10) at 0, 1, 10, 25, 50, and 100 ng/mL, respectively. *Panel B*, *lanes 1-6*: cells treated with glucose at 0, 5.5, 10, 20, 30, and 40 mmol/L, respectively. GAPDH: anti-glyceraldehyde-3-phosphate dehydrogenase.

Western blot analysis was conducted to examine the effects of IL-10 and glucose on OPG and RANKL protein expression (Figure 3). Table 3 presents the densitometric analysis of Western blots. Similar to the mRNA findings, high IL-10 and glucose concentrations had opposing effects on the protein expression of OPG and RANKL.

**Table:**
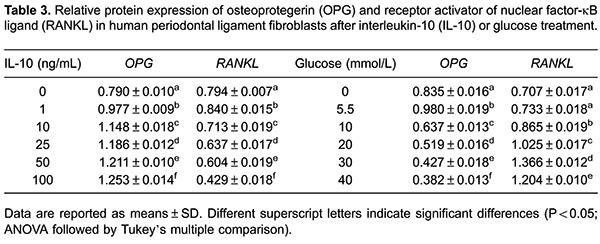

## Discussion

Periodontal disease and diabetes are both prevalent disorders (14). Epidemiological studies indicate that periodontal disease and diabetes share some common risk factors, and represent high risk factors for each other (15). The main biochemical characteristic of diabetes is elevated glucose levels, which play a significant role in the initiation and progression of this disease. High glucose levels have also been demonstrated to increase osteoclast activity, accelerate bone resorption, and cause aberrant bone metabolism (9). There is a close relationship between glucose levels and the progression of periodontal disease (16). OPG has the ability to inhibit osteoclast differentiation and bone resorption, and induce apoptosis of mature osteoclasts (17). Accordingly, OPG knockout mice display a severe reduction in bone mineral density because of osteoclast activation and increased bone resorption (18), whereas OPG transgenic mice exhibit an increase in bone mineral density (19). RANKL promotes bone resorption by enhancing osteoclast motility and inhibiting apoptosis (20). Under normal physiological conditions, OPG is expressed more highly than RANKL in HPDLFs, which promotes stabilization of the periodontal tissue (21). In periodontal disease, the expression of RANKL increases relative to that of OPG, resulting in periodontal tissue destruction (22,23). Data from the present study demonstrate that above-physiological glucose levels induced significant changes in OPG and RANKL expression in HDPLFs. Upregulation of RANKL under high glucose conditions has also been described in human periodontal ligament cells (24). García-Hernández et al. (9) reported that glucose stimulation increased mRNA expression of RANKL and decreased mRNA expression of OPG in human osteoblastic cells. These findings may provide an explanation for exacerbation of periodontal disease by diabetes-associated hyperglycemia.

The pathogenesis of diabetes is associated with changes in the production of anti- and pro-inflammatory cytokines. IL-10, as a pivotal anti-inflammatory cytokine, is usually downregulated during the development of diabetes (10). IL-10 has the ability to downregulate the synthesis of pro-inflammatory cytokines, including tumor necrosis factor-α (TNF-α), IL-1β, IL-6, and IL-8 (25). IL-1β has been shown to upregulate RANKL expression in human periodontal ligament cells (26). The combination of TNF-α and IL-6 has been reported to induce mouse osteoclast-like cells with bone resorption activity (27), suggesting that IL-10 has a favorable role in bone formation. Indeed, IL-10 knockout mice had significant alveolar bone loss compared with wild-type mice (9). In the present study, IL-10 caused concentration-dependent upregulation of OPG expression in HPDLFs. Conversely, RANKL expression was concentration-dependently reduced in IL-10-treated HPDLFs. These results confirm the involvement of IL-10 in bone remodeling pathways. A previous study reported that injection of IL-10 to HuPBL-NOD/SCID rats infected by *Actinomyces* resulted in significantly less alveolar bone resorption (28). Local delivery of exogenous IL-10 may represent a potential treatment for periodontal disease.

This study has a few limitations. Of note, the signaling pathways that mediate regulation of OPG and RANKL by glucose and IL-10 remain to be clarified. In addition, the combined effect of glucose and IL-10 on expression of OPG and RANKL is not known.

To conclude, high concentrations of glucose upregulated RANKL and downregulated OPG, whereas IL-10 produced opposing effects to those of high glucose in HPDLFs. These findings warrant further investigation of the effect of glucose on the expression of OPG and RANKL, and on bone remodeling in periodontal disease.
